# Improving the Fit of Interventions for Autism in Schools: Lessons Learned Following Paraeducator Coaching on the RUBIES Program

**DOI:** 10.1080/10474412.2025.2578840

**Published:** 2025-11-05

**Authors:** Daina M. Tagavi, Sydney Puga, Catherine Dick, Angela V. Dahiya, Karen Bearss, Jill Locke

**Affiliations:** a University of Washington, Seattle; b Seattle Children’s Hospital; c Psychiatry, Kaiser Permanente Autism Spectrum Disorder Center; d Catalight Foundation; e University of California, Los Angeles

## Abstract

Implementing evidence-based behavioral interventions in school settings presents various challenges, particularly for paraeducators who often have limited formal training in working with autistic students, let alone specific behavior management strategies for this population. This study examined paraeducator feedback on the RUBI in Educational Settings (RUBIES) intervention, an adapted evidence-based behavioral intervention for schools. Sixteen paraeducators who participated in a randomized controlled trial of RUBIES completed semi-structured interviews, providing insights into the program’s strengths and areas for improvement. Using the Framework for Reporting Adaptations and Modifications – Expanded (FRAME), feedback was categorized into (a) positive feedback and (b) adaptation suggestions in the areas of (a) content and (b) implementation and scale-up activities. Paraeducators found RUBIES content effective in reducing challenging behaviors and improving student engagement. However, implementation barriers such as scheduling constraints, difficulty accessing materials, and limited coordination with broader educational teams were identified. Recommendations included expanding staff training and integrating digital tools for real-time strategy use and data collection. Findings highlight the need for ongoing monitoring and iterative adaptation of RUBIES implementation strategies in collaboration with community partners to ensure its feasibility and sustainability in school settings. Future research should focus on refining RUBIES and assessing long-term outcomes to enhance the program’s impact on paraeducators and autistic students.

## Introduction

Autism is becoming increasingly identified in the United States, with 1 in 31 children currently diagnosed (Shaw et al., 2025). Autistic students, relative to non-autistic peers, exhibit more frequent challenging behaviors, including aggression, self-injury, elopement, and transition difficulties (Kaat & Lecavalier, 2013; Lory et al., 2020; Simó-Pinatella et al., 2019), which have been shown to impede both positive academic and social functioning in the school environment (Warren et al., 2006; Waters & Healy, 2012). Additionally, challenging behavior has been reported by teachers of autistic children to be one of their most concerning issues (Azad & Mandell, 2016). Current intervention models for school behavioral challenges rely on highly specialized staff (i.e., special education teachers, board certified behavior analysts, school psychologists) to develop individualized behavior plans, which is both time-intensive and costly. Further, these school staff with specialized expertise often are not providing the day-to-day behavior support for autistic students. Instead, staff with the most direct role supporting autistic students (for example, paraeducators) are least likely to have formal training in evidence-based practices for behavior management in autism (Massafra et al., 2020; Morin et al., 2021). Providing paraeducators with specialized training in evidence-based behavioral management strategies for autistic children can strengthen their ability to effectively manage challenging behaviors, leading to improved student participation in inclusive settings and a more supportive classroom environment overall (Sam et al., 2023).

Although evidence-based practices (EBPs) are essential in school settings, their implementation often is challenging, as many interventions are not designed with educators’ needs in mind (Azad et al., 2021; Pellecchia et al., 2018). Variability in classroom resources, educational materials, and staffing constraints can lead to inconsistencies in implementation, while the demands of daily school routines often limit educators’ capacity to use these practices consistently. Additionally, factors such as small staffing ratios and a lack of specialized training can make it challenging to implement structured interventions in schools (Locke et al., 2015, 2017). To bridge this gap, schools require contextually appropriate interventions that are practical, adaptable, and feasible for educators to apply in real-world settings.

The Research Units in Behavioral Intervention (RUBI) parent-mediated intervention has a robust evidence base for reducing co-occurring challenging behavior and increasing adaptive skills in children ages 3 to 14 with autism and/or other developmental disabilities (Aman et al., 2009; Bearss et al., 2015; Handen et al., 2015). RUBI is a manualized, 11-session program that is designed to be delivered via weekly hour-long sessions in outpatient settings in order to teach caregivers how to: (1) understand behavior through the lens of autism, (2) use a behavior analytic framework to identify function(s) of behavior, and (3) systematically and strategically apply a range of behavioral strategies directly with the child in their naturalistic environment based on this functional assessment (Bearss et al., 2018).

Given the need for low-intensity, evidence-based interventions to address challenging behaviors for autistic students in schools, RUBI was adapted for use in educational settings. RUBI in Educational Settings (RUBIES) was developed through an iterative re-design process of RUBI using comprehensive input gathered from school partners (Bearss et al., 2022). This adaptation was focused on tailoring RUBI content to be more practical and accessible for providers working in schools. This included modifications to intervention content to fit the needs and priorities of public schools, and identifying the end-user of RUBIES. Content modifications included: (1) incorporating school-specific examples, (2) adding an “Autism 101” psychoeducation module, (3) providing a visual toolkit to simplify the creation of highly valued visual supports, (4) merging the two reinforcement sessions into a single session, (5) redesigning compliance training to emphasize proactive support with instructional follow-through, (6) integrating planned ignoring techniques with functional communication strategies, and (7) removing sessions focused on teaching skills (Bearss et al., 2022). Beyond content changes, identifying the ideal end users of RUBIES was a key part of the redesign. Special education teachers felt confident using behavioral strategies but could not follow students across general education settings (Bearss et al., 2022). General education teachers reported needing training in EBPs for autism (Tagavi et al., 2024), however, they face barriers to accessing trainings and using EBPs such as time constraints and large class sizes (Harbin et al., 2024). Meanwhile, paraeducators, who spend the most time with autistic students, reported little formal training in autism and behavioral strategies (National Resource Center on Paraeducators, 2023). Given their direct and ongoing student interactions, paraeducators were identified as the primary end users of RUBIES (Bearss et al., 2022).

In addition to RUBIES content, several implementation strategies, methods or techniques used to enhance the adoption, implementation, and sustainability of a clinical program or practice (Curran et al., 2012), were designed as part of the original program to support the use of RUBIES in schools. The initial RUBIES implementation strategies that complemented RUBIES content included: 1) practice check-ins (or fidelity review of paraeducators’ use of strategies), 2) didactic instruction of RUBIES content via 9 weekly, 60-minute Zoom meetings with a RUBIES coach (however, this is individualized based on educator need/preference; See Table 1 for a breakdown of the typical session structure for RUBIES), and 3) practice planning of behavioral strategies to use with the autistic student, via weekly individualized sessions with a RUBIES coach. These are aligned with implementation strategies that were identified as both highly important and highly feasible among a set of implementation strategies adapted for the school setting (for example, conducting ongoing training, providing ongoing consultation/coaching, and monitoring progress of the implementation effort; Lyon et al., 2019), however, these strategies were not explicitly modified in the original redesign efforts.

While community partner feedback was integral to the initial RUBIES re-design, an important element of intervention adaptation is employing a continual iterative improvement process to make updates based on feedback from users to enhance the fit, and thus effectiveness and sustainability, of an intervention when delivered in a new context (Chambers & Norton, 2016). The purpose of this study was to analyze exit-interview feedback from a subset of paraeducators who participated in the initial randomized controlled trial of the RUBIES intervention to achieve two primary goals: 1) to evaluate strengths and areas for improvement in RUBIES content; and 2) to assess how RUBIES’ implementation strategies worked for paraeducators in the school setting. These findings will directly inform further adaptations to both RUBIES content and implementation strategies. Additionally, this study provides an example of how community partner feedback can be solicited, examined, and quickly integrated to improve subsequent adaptations of an intervention and implementation strategy to support use and improve outcomes for autistic students.

## Method

### Study design

This study was part of a larger randomized controlled trial (RCT) that examined the impact of RUBIES on autistic students’ challenging behavior in public school settings compared to a psychoeducation control condition (Bearss et al. 2025). Random assignment was completed by a research project coordinator in RedCap and occurred at the paraeducator level (with each paraeducator paired with a target student with whom they would implement RUBIES). Randomization was stratified according to two factors: (1) student grade level (Kindergarten – 2nd and 3rd – 5th grade) and (2) the proportion of time spent in inclusive settings (≥50% and < 50%). This study was approved by the Seattle Children’s Research Institute Institutional Review Board (STUDY00001890) and was pre-registered in the clinical trials database (clinicaltrials.gov; identifier NCT05093686).

### Participants

Sixty-seven paraeducators enrolled in the larger RCT. Inclusion criteria for paraeducators included: (a) employed as a public school district paraeducator in an elementary school, and (b) supported a consented autistic student with mild to moderate challenging behaviors (defined as teacher or paraeducator ratings on the Aberrant Behavior Checklist-Irritability (ABC-I) subscale (raw score ≥10) and/or the ABC-Hyperactivity (ABC-H) subscale (raw ≥20)) with whom they would be willing to implement RUBIES. Thirty-four paraeducators were randomized to the RUBIES condition and thirty-three paraeducators completed RUBIES (one paraeducator was unresponsive to attempts to schedule RUBIES sessions); all paraeducators who completed the RUBIES training and used RUBIES with their identified student were invited to participate in the follow-up interview. Sixteen paraeducators (48.5%) opted in to complete an interview. Interviews were an optional component at the end of the study, and it was not expected that all paraeducators would opt in. Paraeducators were employed by 16 different schools in seven states across the US. See Table 2 for paraeducator characteristics.

### Measures

#### Semi-structured interview guide

The interview guide (Appendix A) was designed by the principal investigators of the study (last two authors) to elicit paraeducator feedback on the RUBIES content, the implementation process, and the generalizability of the program to other students and educators. The interviews followed a funnel approach (Spradley, 2016), with broad inquiries followed by specific follow-up questions in these three areas.

#### Procedures

Following the completion of RUBIES, paraeducators were invited to participate in the qualitative interview. Interviews were conducted via Zoom by four research assistants who were trained in qualitative interviewing skills by one of the study Principal Investigators, a mixed methods expert. Interviews lasted 45–60 minutes and participants were given a $50 gift card for their time.

#### Qualitative data analysis

Our team utilized the Framework for Reporting Adaptations and Modifications – Expanded (FRAME; Wiltsey Stirman et al., 2019) to conceptualize and organize qualitative data. The FRAME is a structured tool designed to guide the systematic identification, categorization, and reporting of adaptations and modifications made to evidence-based interventions. Originally developed within implementation science, FRAME was designed to improve transparency and consistency in documenting how interventions are changed when moved into new settings, populations, or delivery systems. It has been applied across a range of health and behavioral health interventions to capture not only what was modified, but also the reasons for modification and the decision-making processes underlying these changes (Lau et al., 2017; Miller et al., 2021; Rabin et al., 2018). FRAME distinguishes between planned and unplanned adaptations, categorizes changes as content- or context-related, and considers their impact on intervention fidelity and outcomes. Importantly, FRAME also identifies who participated in the decision-making process to modify the intervention, providing critical insight into the collaborative dynamics of adaptation (Wiltsey Stirman et al., 2019).

We selected FRAME to conceptualize and organize our study because it provides a comprehensive approach to categorizing adaptations, capturing both the nature of the changes and the decision-making process. FRAME provided an ideal structure for organizing our data, allowing us to systematically categorize paraeducators’ feedback into positive and constructive insights within the domains of (1) RUBIES content adaptations and (2) implementation strategy adaptations. Positive feedback emphasized the importance of maintaining fidelity to the core components of RUBIES, such as the effectiveness of its behavioral strategies, which paraeducators found practical and impactful. In contrast, constructive feedback highlighted specific areas where modifications could improve the intervention’s fit and usability.

All semi-structured interviews were digitally recorded and transcribed via an online transcription service. Rapid qualitative assessment methods were used to analyze the interviews (Hamilton, 2013; Hamilton & Finley, 2019). We selected a rapid qualitative analysis approach to allow for timely, iterative refinement of the adapted intervention. Given the need to make quick modifications following participant interviews and prior to re-implementing the intervention in subsequent testing cycles, this method allowed us to efficiently identify and respond to implementation barriers and areas for improvement. The structured nature of rapid qualitative methods enabled us to synthesize feedback quickly while maintaining momentum in the adaptation process. While more traditional qualitative methodologies offer greater depth and theoretical abstraction, rapid analysis provided timely, actionable insights that aligned with our applied goals and community partner-engaged approach. This method has been found to yield comparable findings to other established qualitative approaches, such as thematic analysis (Gale et al., 2019; Nevedal et al., 2021; Taylor et al., 2018).

The lead author created a neutral domain name that corresponded with each interview question and drafted a “summary template” to organize responses to interview questions under corresponding domain names. The lead author then trained coders to document key statements from the interviews through a minimally interpretive process. Next, the team (consisting of the lead author and two postdoctoral fellows) piloted the template with a subset of transcripts to ensure that the domains were intuitive for the data before summarizing the remaining transcripts (Hamilton, 2013). One third of the transcripts were double coded and inter-rater reliability was high (> 90%). Finally, the summaries were transferred to a matrix (respondent by domain) to facilitate the process of systematically noting similarities, differences, and trends by respondents (Averill, 2002) and to generate themes (Harkness et al., 2022). We achieved data saturation in our interviews, as no new themes or insights emerged in the later interviews, indicating that additional data collection was unlikely to yield novel findings. Finally, themes from the matrix were sorted into FRAME domains.

## Results

### Paraeducator feedback on RUBIES content

See Table 3 for themes that emerged during the coding process as well as resulting adaptations made by the research team to the RUBIES content and implementation strategies to address constructive feedback.

#### Positive feedback

Feedback from paraeducators highlighted several strengths of the RUBIES intervention content. Specifically, coding revealed positive feedback regarding RUBIES content in five areas.

##### RUBIES strategies are usable and effective.

Paraeducators consistently reported that specific strategies within RUBIES, such as planned ignoring, visual timers, visual schedules, creative token systems, and replacement behaviors, were both usable and effective in their school settings. They noted that these strategies were instrumental in both decreasing challenging behaviors and increasing positive behaviors among students. For example, one educator stated:

I learned strategies that I’m using daily that are super helpful for me and my student. The visual timers and visual schedule, just to manage that anxiety about what’s coming next – those are the things that I really use a lot. And then also the tokens - popcorn points is what we’re calling them.

Another paraeducator recounted:

The planned ignoring was something that worked great. So instead of feeding into the tantrum … [knowing] it’s attention seeking behavior. Me and the teacher worked so well together. When he was having those moments, we would look at each other and go oh, planned ignoring, and allow him to have his moment. Those moments would go by so much faster, and they’d end so much quicker if we didn’t engage.

##### RUBIES strategies are broadly applicable.

Paraeducators reported that they appreciated the adaptability of the intervention, emphasizing that the content could be individualized to address specific student needs. Additionally, several paraeducators stated that they appreciated the breadth of strategies to target different types of student behaviors. For example, one paraeducator recounted:

I liked having multiple ways to reinforce or change or maintain behaviors … It was like, oh, that didn’t work, so let’s try this or let’s add this in … there were lots of options to pick from for the different behaviors.

Similarly, participants stressed that RUBIES was broadly applicable across various student populations, including neurotypical peers, which greatly increased their confidence in their jobs overall. For example, one paraeducator said:

I definitely have used RUBIES with other students and not just my focus student. It gave me a lot of ideas of ways I could tweak little things that I do and say with all the kids that I work with, so I really feel like I’ve grown professionally that way. I have a lot of new tools in my belt, and I know how to use them, I know why I’m using them, and I know how they’re going to benefit my students. I felt like the things in the training gave me ideas on how to manage almost any behavior that we could come across.

Similarly, another paraeducator recounted:

I’ll use these strategies, not just with kids on the autism spectrum. I use these same strategies with kids who just have behavior issues. Because there might be some differences, but behavior is behavior. And it has a function, and it has a purpose, and I think it is just really good to bring it back to not so much looking at the behavior, but what is the function? What is the child trying to communicate? And how can we shape this to a healthier way of getting to what they want or need or replacing or fixing it?

##### RUBIES improved target student relationships.

Paraeducators also highlighted the positive ripple effects of RUBIES strategies, such as improved peer responsiveness and empathy toward autistic students. For example, one paraeducator reported:

Her peers are extremely patient … students in the whole class now understand she doesn’t want them to bring things when she forgets. She’s pretty independent. And classmates are learning that they need to give her the opportunity to do things by herself. I think [RUBIES] is all very positive and that [the students] also have the opportunity to learn.

Many participants also reported increased confidence, patience, and rapport with their students as a result of implementing RUBIES strategies. For example, another paraeducator explained:

It helped me be more patient and see things from a different perspective. Seeing things more from their side, so this [behavior] is calling for something that they just don’t know how to ask for, so let’s step back and try to figure out what’s going on.

### RUBIES visual kit helpful

Paraeducators consistently reported that the RUBIES visual kit, provided by the research team, was a particularly helpful component of the intervention. They described the visuals as easy to use, engaging for students, and effective in supporting behavior and communication across various classroom situations. Several paraeducators noted that the visuals enhanced their ability to implement strategies consistently, even during challenging moments. One participant shared:

We did star charts, which were just excellent. And they have spread around the whole room. I started with the five-star star chart, which was provided. It was fantastic. And when a behavioralist came in and said, well, it would be nice if you could have more stars. Well, I’ll be darned, there’s a ten-star chart in there, so I switched to that. And the icons that are provided give you ideas on what could be things they’re working towards.

Similarly, another paraeducator another remarked, “Transitioning him from a token board to a first-then board, that was huge for him because it made it feel like he had a little bit more control, as he was really starting to understand.” These findings underscore the value of having concrete, ready-to-use visual tools that can be quickly integrated into daily routines.

#### Constructive feedback

Paraeducators also identified five broad areas for improving RUBIES content.

##### Content difficult to generalize to other environments.

Participants expressed a need for tools to help generalize strategies to other environments, such as the home. For example, one paraeducator recounted, “I would have a strategy in place, and it would be working Monday through Friday and then Monday is a re-start, because home doesn’t have that. So, if families had the same information as we have, that would definitely benefit.” Several paraeducators observed that difficulties across settings sometimes contributed to behavioral cycles in which students returned to school dysregulated, making it challenging to maintain progress from day to day. For example, another paraeducator noted:

And then just like getting him to be in school was difficult. His parents were very understanding in the beginning, but then as his behavior started becoming worse, it was just harder to get them to implement some of the strategies that we were trying to do at school at home.

Additionally, paraeducators reported that content was also difficult to generalize within the school environment, such as between classrooms or school activities. One paraeducator stated, “It’s sometimes hard, because with my student, when she’s in a general education environment, and there are many, because she goes to her different specialists, and there’s a different one every day, and they all have different expectations.”

##### Manual materials not representative.

There also was feedback given regarding the limited range of examples in the materials, which did not adequately cover all developmental levels, cognitive abilities, or language proficiencies. One paraeducator recommended, “ … if you had something with bigger kid icons on it, maybe a football player or having something that before you create it, just ask the level of the kid and then those icons can match their developmental level.” Similarly, another paraeducator shared, “It just feels like it’s definitely geared for early childhood or non-verbal kids. If you edit different examples in [the] manual, I could see the potential of [the strategies] helping high schoolers as well.”

##### Self-care and self-regulation not addressed.

Paraeducators also suggested adding content on self-regulation and self-care for educators, noting the stress associated with managing challenging behaviors:

I know that we talked a lot about how to help de-escalate and regulate our students, but there are a lot of times that I would struggle with being escalated because my student was escalated, or there was another student that was escalated that I was trying to assist with and trying to find that balance to help regulate ourselves. So, if you had a small section focused on the paraeducators, to help them with self-care, that would be cool.

Relatedly, another paraeducator expressed:

I would love info on how to communicate with [students] while at the same time trying not to match their energy, because that’s the struggle. What I see a lot, especially around March or after spring break, we’re at our realm where we’re stressed waiting for kids to explode. So, it would be great to have something to assist us with keeping our body calm.

##### Difficulty navigating RUBIES concrete materials.

Some challenges were also noted with the RUBIES workbook and visual kit, particularly in navigating and locating specific content or terms. Recommendations included adding a “glossary of terms” to help prime educators for what is to come as well as help them easily refer back to content they are looking for in the workbook, “separating data collection pages from the workbook” to make collecting and referring back to data easier and faster, and creating an “instructions page for the visual kit” to help guide the selection of visuals in various situations. Additionally, one paraeducator recommended some smaller visuals for easy transportation:

You know what might be great, because I’m that person, I’m going in and out of classrooms. If you had a small, a pretty small chart, something you can keep in your pocket to show a student. When you’re in the gen ed setting.

Constructive feedback from paraeducators directly informed several revisions to the RUBIES content. In response to their input, the research team added a specific goal to each session in the RUBIES manual focused on promoting generalization across settings. The manual was also enhanced with more diverse examples and increased time for problem solving during coaching sessions to support the adaptation of strategies for students with varying developmental needs. To improve usability, each session now includes a summary handout highlighting key terms. Additionally, an overview page was added to the visual kit to guide educators on when different visuals are most commonly used. Although educator well-being emerged as an important theme, incorporating additional content on self-care for educators was outside the scope of the current redesign.

### Paraeducator feedback on implementation strategies

#### Positive feedback

Paraeducators also provided valuable insights into the implementation of RUBIES within their school setting, identifying both strengths and challenges.

##### Appreciate RUBIES coaching structure.

The structure of RUBIES training received positive feedback, with paraeducators appreciating its scaffolding of skills, real-life examples, and built-in time for review. For example, one paraeducator stated, “I was glad that it was scaffolded in that we started with one thing and then that worked well, okay, so now we’re gonna add this.” Another paraeducator noted:

It was nice for me to have like this brush up, and then also to talk it through and having an opportunity to say like, okay, so I tried this, and it didn’t really work, or it kind of worked, or it worked on Monday and didn’t work on Wednesday and things like that. Like it was really nice to have a chance to like riddle it out.

Although paraeducators noted that strategies were applicable to a wide range of students, several participants also expressed an appreciation for taking the time to focus in on just one of their students:

It was really nice to be able to talk also about the one particular student that we were focusing on and to make different correlations with the training information and his behaviors and his own personal circumstances and kind of see how it all tied together.

##### Appreciate diversity of content delivery options.

The ability to access content in various formats was also viewed as a significant strength, supporting diverse learning preferences. As an example, one paraeducator said:

I really liked that there was a lot of information given, and that we had different ways to access that information. We had face-to-face weekly coaching, but then we also had concrete materials that were sent to us, like the visuals and the printed booklet. And then, we also could access online and print from there if we needed to, or just use it online. And so, I liked having multiple ways to access because sometimes I might forget something, and I always had access.

Consistent with this, another paraeducator reported, “The way I received the information was … very direct … I liked the having the concrete materials to fall on with what [coach] was talking about. I just liked having the resource – just the box of [visuals] and the workbook.”

#### Constructive feedback

Implementation challenges primarily stemmed from logistical and systemic factors. Specifically, five broad themes were noted in this area.

##### Scheduling difficulties.

Paraeducators reported difficulties scheduling RUBIES sessions due to the dynamic and often unpredictable nature of school environments. Staffing shortages and limited one-on-one time with students were also significant barriers. One paraeducator gave the following feedback:

A couple of those challenges included him not being in school, us not having enough prep time, enough staff to really work with him individually. And for the most part, I was able to recognize where those behaviors and patterns are coming from, but being able to apply the strategies to be able to change those behaviors was where it became difficult.

Other paraeducators felt compelled to complete the training outside of their work hours, which proved challenging. For example, one paraeducator commented:

It was hard with scheduling to do something that was synchronous. I think it would have been easier if I could have scheduled it during my workday. I think if I participated in something like this again, I would ask admin to let me have a specific time during my workday to do it.

##### Lack of broder team knowledge about RUBIES and student goals.

Additionally, several paraeducators highlighted a lack of awareness among broader educational team members regarding student goals and RUBIES strategies, which limited consistency and generalization. Most paraeducators recommended that RUBIES more explicitly train or include other teachers or administrators in the program. For example:

It would be great if there was more communication between team members, because we all work with these students, and if I try some things and they’re successful or not, if I don’t share that information with others that work with the student, they’re not going to be getting reinforced throughout the day or generalizing to other settings. And most people are willing to follow a behavior plan if they’re made aware of what’s going on, and so, sharing information is really important.

Another paraeducator shared similar sentiments:

It could be very useful to have other RUBIES trained people in the school, other paras like me, or teachers that can collaborate, kind of, or bounce ideas off of since I was the only one at the time doing the study in my school, It would be nice to have a different person who knows the same methods, the same strategies, the same kind of ideas to say, hey, maybe I’m struggling with this particular behavior, I’m thinking about this, what do you think – kind of collaborate with other people.

Paraeducators also gave the feedback that it could be helpful to have longer-term check-ins with others familiar with RUBIES to ensure they are using RUBIES with fidelity: “Maybe if there were [continued check-ins], like a quarterly check-in where I could report in and troubleshoot. Something like that would be helpful.”

##### Confusion surrounding integrating RUBIES with others EBPs.

Paraeducators also suggested that coaching sessions should intentionally integrate existing evidence-based practices or strategies on students’ IEP or behavioral plan to ensure consistency. One paraeducator explained: “So I don’t know how you make it more known to the special ed departments, to the principals … because it complements a lot of what we’re doing [on the students’ plans]. So yeah, just like streamline things, I guess.” Aligned with this perspective, another paraeducator explained, “I think RUBIES can investigate what we are learning from the state mandatory training and maybe see which area paras are having the most difficulties and develop more information in those areas, so [the programs] complement each other.”

##### Materials difficult to use/reference in the moment.

Study participants also reported specific challenges using RUBIES materials and tools within the school setting. For example, some paraeducators expressed dissatisfaction with the formatting of the RUBIES workbook, stating that they wish it had a separate data collection pages so they did not have to carry their workbook around with them at all times of the day. Additionally, study participants found it difficult to collect behavioral data. One paraeducator stated, “Taking data for things was a challenge. We’re very busy during the day at school, and I would sometimes not have the sheets I needed to do the data – I would leave it at home or leave it in another [classroom].” Paraeducators expressed a need for resources that are easier to reference in real-time, such as a portable or digital tool like a mobile app, that could be used to collect data and review RUBIES strategies:

I’m thinking an online interface might have been helpful just for me to pop on, like an app because I always have my phone with me … an app where I could get in there and either put the information in so that it was real time. And being able to look at a couple of the strategies and a few key words.

Several paraeducators also suggested that additional online tools could include both (a) video recording of coaching sessions and (b) example videos of an educator using strategies in real time. For example, one paraeducator stated, “I think a video example of what planned ignoring might look like, that would be brilliant. And it’s something I could show another staff member too. A short video for each of the strategies could be super helpful.” Similarly, another paraeducator expressed, “I know my meetings with [my coach] were recorded, and sometimes it would have been nice to have those so I could relisten if I wanted to because there were some nuggets that I didn’t even write down and it just happened as we were processing and dialoguing.”

Paraeducators’ feedback also informed several refinements to the RUBIES implementation strategies to enhance feasibility and alignment with school practices. To improve accessibility, more flexible scheduling options were offered at the onset of RUBIES. A new Collaborative Orientation and Treatment Planning Session was added, allowing the RUBIES coach to meet with the student’s broader educational team to provide an overview of the intervention and develop a plan for consistent communication of strategies across team members. Coaches now also collect and review the student’s Individualized Education Program (IEP) and Behavior Intervention Plan (BIP) during the first coaching session to better align RUBIES content with existing supports. Finally, a mobile application was introduced to provide educators with a built-in data collection tool and easily accessible review materials for RUBIES strategies.

## Discussion

This study aimed to gather iterative qualitative feedback on RUBIES content and implementation strategies following an initial redesign study and pilot test with paraeducators working with autistic students with challenging behaviors in public schools. We utilized the Framework for Reporting Adaptations and Modifications-Expanded (FRAME) to organize feedback, and paraeducators insights broadly fell into four areas: (1) positive and (2) constructive feedback regarding (1) RUBIES content and (2) RUBIES implementation strategies. Findings from this study led to key adaptations to the RUBIES content and implementation strategies, including the addition of goals focused on generalization, diverse examples and session handouts, a collaborative orientation session, alignment with IEPs and BIPs, and a mobile application to support strategy use and data collection. Although explicit content related to educator self-regulation was not incorporated, the modifications intended to enhance usability and contextual fit of the intervention may indirectly bolster educators’ self-efficacy, and therefore emotional regulation, during behavioral challenges.

Importantly, paraeducators identified several implementation strategies that they found especially supportive of fidelity and usability. They valued the scaffolded coaching structure, which provided opportunities for guided practice, problem-solving, and individualized application to a focal student, approaches that reflect evidence-based methods in coaching and ongoing consultation (Kretlow & Bartholomew, 2010; Reddy et al., 2022; Snyder et al., 2015). They also highlighted the availability of multiple formats for accessing RUBIES content (e.g., coaching, printed and visual materials, online access) as a major strength, underscoring the importance of flexible, multimodal supports for sustaining implementation in schools (Hugh et al., 2023).

Our findings align with broader literature on adapting EBPs for schools, underscoring the need to adapt both the “what” of EBPs (e.g., RUBIES content) and the “how” it is implemented (e.g., RUBIES implementation strategy) in resource-constrained environments (Harn et al., 2013; Lyon & Bruns, 2019). Similar to prior research on school-based behavioral interventions (Eiraldi et al., 2015; Harbin et al., 2024; Locke et al., 2015, 2019; Suhrheinrich et al., 2021), paraeducators faced challenges integrating RUBIES into daily routines due to limited support from colleagues and logistical challenges completing components of RUBIES (e.g., data collection, easily accessing review materials). RUBIES coaches, as well as other school-based consultants hoping to implement EBPs, can take several steps can enhance the usability and generalizability of RUBIES and similar school-based interventions. Professional development workshops for entire school teams, not just paraeducators, could promote shared ownership and consistency of behavioral strategies (Bateman et al., 2022; Ryndak et al., 2021). Additionally, developing scalable digital tools, such as mobile applications, could improve access to strategies, support data collection, and enhance long-term fidelity and implementation of RUBIES (Merlo et al., 2018; Wainberg et al., 2021). To improve flexibility and fit within existing school structures, coaches can work with educators to align RUBIES strategies with other behavior support plans and frameworks already in use (e.g., PBIS, MTSS, or individualized BIPs), helping to streamline efforts and reduce redundancy. Embedding RUBIES coaching within existing meeting structures or classroom routines can also reduce burden and support sustainability, particularly when paired with flexible scheduling options that accommodate paraeducators’ varied roles and availability throughout the school day.

Given the unique nature of school settings, adaptation of EBPs for this context must be an ongoing process, requiring continuous and iterative redesigns and engagement with community partners (Aarons et al., 2019; Cook et al. 2019). A fundamental component of implementation practice is selecting and adapting implementation strategies to address the specific barriers within a given service setting (Powell et al., 2017). While many implementation strategies are broadly applicable across various contexts, the education sector presents unique characteristics, which influence the relevance and suitability of specific strategies (Lyon et al., 2019). In response to what was learned from this qualitative study as well as the larger RCT, further modifications have been made to both RUBIES content and implementation strategies to ensure a more seamless integration within educational environments. Additionally, following this study, our team conducted a systematic redesign of the RUBIES implementation strategies via focus groups with community partners (i.e., school administrators, teachers, paraeducators) and are currently in the process of testing a redesigned suite of RUBIES implementation strategies.

Building on the positive feedback provided by paraeducators, we retained elements of RUBIES that were especially well-received, such as scaffolded coaching, individualized focus on students, and multiple formats for accessing content. To address systemic barriers to RUBIES implementation, we have adapted the intervention to better align with existing educational structures, such as individualized education plans (IEPs), behavior plans, 504 plans, and academic curricula, taking care to ensure that RUBIES is embedded within a school’s broader student support system. These adaptations aim to enhance consistency, reduce fragmentation, and create a more cohesive approach to addressing both behavioral and academic needs. Additionally, we have integrated tools for real-time data collection and in vivo learning, such as mobile applications, video examples of strategy implementation, and online portals with reminders to practice RUBIES strategies, to support paraeducators in tracking progress and refining their use of RUBIES strategies. To further strengthen implementation, we are currently testing a team-based version of the RUBIES implementation strategy that actively engages members of the student’s broader educational team, including teachers and administrators. This approach is designed to promote consistency across educational staff, facilitate generalization of RUBIES strategies across different settings, and enhance long-term sustainability. We hope that this systematic and iterative approach to redesigning and adapting RUBIES ensures that the intervention is not only effective in the short term but also capable of driving lasting improvements in paraeducator practices and student outcomes.

### Limitations

While overall findings provide clarity on ways to improve and adapt RUBIES content and implementation, there are several limitations. This study focused on implementation of RUBIES with paraeducators in public elementary schools. Findings from this study may not be applicable for educators in different roles (e.g., specialists, general education) and/or providers in other settings. Additionally, demographic variability was limited, which in turn, limits the applicability of RUBIES implementation within more diverse populations. Moreover, since only about half of the invited paraeducators (16 of 33) participated in the interviews, there is a potential for self-selection bias. This bias may lead to an overrepresentation of those who felt more confident in their implementation abilities or were more engaged with the program, potentially skewing feedback toward more favorable perspectives. As a result, the experiences of paraeducators who faced greater challenges in implementing RUBIES or dropped out may be underrepresented, potentially overlooking key barriers to effective implementation. The parent RCT of this study was conducted during the height of the COVID-19 pandemic, a period that significantly disrupted the school environment and may have had downstream effects on RUBIES implementation, ultimately shaping paraeducators’ qualitative feedback.

The feedback collected in this study was gathered shortly after paraeducators’ completion of RUBIES training, reflecting participants’ immediate reactions and experiences. As a result, the findings do not capture paraeducators’ ability to sustain or generalize the use of RUBIES strategies over time. Longitudinal research is essential to assess whether the reported outcomes persist and to identify any additional challenges or areas for improvement that may arise with long-term implementation. Additionally, this study relied exclusively on data from paraeducators and did not include perspectives from other key educational partners, such as teachers, administrators, or parents. Incorporating these viewpoints could provide a more comprehensive understanding of implementation barriers and facilitators. Including these voices could inform potential changes for effective training and support strategies to be modified for the school environment given the education and care of autistic students are not often conducted in isolation and often requires a multidisciplinary team.

Finally, methodological limitations should be acknowledged, as this study utilized rapid coding to analyze qualitative data to be able to make quick, evidence-based adaptations to RUBIES for future research endeavors. While rapid coding enables efficient data processing (Gale et al., 2019), it may limit the depth and complexity of analysis, potentially reducing the ability to capture nuanced themes. As a result, some intricate participant responses may have been simplified, affecting the interpretation of their experiences. Additionally, we did not engage in member checking, a process where participants review and validate the accuracy of qualitative findings. Without this step, there is a risk that our interpretations may not fully align with our participants’ intended meanings, potentially affecting the credibility of the results.

## Conclusions

This study highlights the importance of continually refining and iteratively testing school-based evidence-based practices to maximize feasibility, usability, and alignment with educator needs. Retaining well-received features, such as scaffolded coaching, individualized focus on students, and multiple modes of content delivery, can preserve what works well in practice. At the same time, expanding training to include broader educational teams and leveraging digital tools for real-time data collection and strategy support can help overcome common implementation barriers. Sustained monitoring and systematic adaptation, informed by ongoing collaboration with educators and school partners, are critical for maintaining responsiveness to evolving classroom needs. Future efforts should prioritize flexible, scalable solutions that align with existing school frameworks, fostering long-term integration and effectiveness in diverse educational settings.

## Figures and Tables

**Table 1.** RUBIES sessions and typical session structure.

Week 1*	Session 1	Autism 101
Week 2	Session 2	Behavioral Principles
Week 3	Session 3	Prevention Strategies
Week 4	Session 4	Daily Schedules
Week 5	Session 5	Reinforcement
Week 6	Session 6	Planned Ignoring + Functional Communication Training
Week 7	Session 7	Compliance Training
Week 8	Session 8	Generalization and Maintenance
Four-Week Break		
Week 12	Session 9	Booster Session

** Note: Typical session structure is one, 60-minute session per week, however, this structure can be individualized based on educator need/preference.

**Table 2.** Paraeducator demographics (N=16).

Paraeducator Characteristic	M (SD) or %
**Age**	44.2 (11.5)
**Gender**	
Female	100.0%
**Race/Ethnicity**	
White	75.0%
Black	6.3%
Asian	6.3%
Hispanic/Latino	6.3%
More than one	6.3%
**Education**	
High school diploma/GED	6.3%
Some college	25.0%
College or advanced degree	68.8%
**Years in position**	3.7 (4.3)

**Table 3.** Qualitative interview feedback and associated adaptations.

Feedback Area		Paraeducator Evaluation	Adaptations Made to RUBIES by Research Team
RUBIES Content	Positive	• Specific RUBIES strategies were usable and effective • Strategies can be individualized based on specific student behaviors • RUBIES content is applicable to other students • RUBIES strategies led to improved understanding of and patience toward target student • RUBIES visual kit usable and helpful	
	Constructive	• Content often difficult to generalize to other environments • Manual written examples and vignettes do not cover all ages, cognitive abilities, or language levels • Difficult to find certain content/terms in workbook • No content on self-regulation or self-care for educators • Challenging to know when to use which visuals from visual kit	• Added specific goal to each session in the RUBIES manual focused on generalization • Added more diverse examples to the manual, as well as incorporated more time for problem solving to adapt strategies for various developmental abilities during coaching sessions • Added summary handout for each session in manual with key terms • *Outside of the scope of this student-focused intervention* • Add overview page for visual kit to describe when various visuals are commonly used
Implementation Strategy	Positive	• Like RUBIES coaching structure • Appreciate being able to access content in various formats	
	Constructive	• Difficult to schedule RUBIES sessions • Difficult to use RUBIES strategies with student due to broader educational team not being aware of student goals/RUBIES strategies • Not clear how to integrate RUBIES with other evidence-based practices being used at school • Materials difficult to reference in the moment throughout school day	• Offer more flexible scheduling options at the onset of RUBIES • Addition of an initial *Collaborative Orientation and Treatment Planning Session*, in which RUBIES coach meets with student’s broader educational team • Collect IEP and BIP from educator and explicitly review documents during first coaching session to allow coach to help better align RUBIES with practices being used in school • Provision of mobile application with built-in data collection tool and review materials covering RUBIES strategies

## Appendices

### Appendix A. RUBIES Interview Guide

There are a number of factors that may impact the use of RUBIES or evidence-based practices for paraeducators or autistic students in schools. We would like to learn from you about your experiences using RUBIES with your included autistic student.

#### RUBIES TRAINING QUERIES

1. What did you like about the RUBIES training overall (these were your weekly meetings with your RUBIES trainer)?
2. What could be improved with the RUBIES training?
3. What topics or content would be helpful to add to the RUBIES sessions?

#### RUBIES IMPLEMENTATION QUERIES

1. Tell me what it has been like for you to use RUBIES for your autistic student.
2. What worked well for you when you used RUBIES?
  How do you feel RUBIES benefitted your student?
  How did using RUBIES strategies benefit you in your role as paraeducator?
  How did using RUBIES strategies benefit the classroom environment?
3. What challenges did you encounter with using RUBIES strategies?
4. What supports would help make it easier to use RUBIES strategies in the future?
  What additional school supports do you wish you had to use RUBIES strategies?
  What would support your continued use of RUBIES strategies after coaching has ended?
5. Tell me how it went with using the tools in the visual kit.

Which visual tools were particularly useful? What would be helpful to add to the toolkit?

Did you share the toolkit with others?

#### RUBIES GENERALIZATION QUERIES

1. Tell me about how it went with using RUBIES strategies with other students (outside of your focal student) in your school.
  Did you use RUBIES strategies with other students (yes/no)?
  If no, what got in the way of using RUBIES strategies with other students?
  If yes, how many other students did you use RUBIES strategies with (ok to ballpark a number)?
  If yes, describe what specific RUBIES strategies you used with other students, including when/how you used the strategies.
2. How did you incorporate information from RUBIES with the student’s team?
  Did you discuss/share information with the team about RUBIES strategies? If so, what information did you share and how was it received by the team?

#### SUMMARY QUERIES

1. What do you think would make RUBIES more useful in schools?
2. What other information would you like to add that I have not asked you today?
